# Lean fish consumption is associated with lower risk of metabolic syndrome: a Norwegian cross sectional study

**DOI:** 10.1186/s12889-016-3014-0

**Published:** 2016-04-19

**Authors:** C. Tørris, M. Molin, M. Småstuen Cvancarova

**Affiliations:** Oslo and Akershus University College, Oslo, Norway; Bjorknes University College, Oslo, Norway; Institute of Basic Medical Sciences, University of Oslo, Blindern, Oslo, Norway

**Keywords:** Metabolic syndrome, Insulin resistance, Diet, Fish consumption, Fatty fish, Lean fish

## Abstract

**Background:**

Fish consumption may have a role in reducing the prevalence of metabolic syndrome (MetS). The aim of this study was to identify associations between fish consumption and MetS and its components, especially regarding differences concerning consumption of fatty and lean fish.

**Methods:**

This cross sectional study uses data from the Tromsø 6 survey (2007–08), where a sample of 12 981 adults, aged 30–87 years (47 % men) from the Norwegian general population was included. Fish consumption was assessed using food frequency questionnaires (FFQ). Blood sample assessments, anthropometric and blood pressure measurements were carried out according to standard protocols. MetS was defined using the Joint Interim Societies (JIS) definition. All tests were two-sided. Analyses were performed using IBM SPSS Statistics 22 (Pearson’s correlation, Chi-Square tests, analysis of variance (ANOVA), linear and logistic regression models).

**Results:**

Mean age was 57.5, and the prevalence of MetS was 22.6 %. Fish consumption once a week or more was associated with lower risk of having MetS among men (OR 0.85, CI 95 % 0.74 to 0.98, *P =* 0.03). In the adjusted models, lean fish consumption was associated with a decreased risk of having MetS, whereas fatty fish consumption was not associated with a decreased risk of having MetS. Both an increased fatty and lean fish consumption (0–1 times per month, 2–3 times per month, 1–3 times per week, 4–6 times per week, 1–2 times per day) were associated with decreased serum triglyceride (TG), and increased high-density lipoprotein cholesterol (HDL-C).

**Conclusions:**

Fish consumption may be associated with a lower risk of having MetS and consumption of lean fish seems to be driving the association. Further investigation is warranted to establish associations between fish consumption and MetS.

## Background

An increasing prevalence of metabolic syndrome (MetS) has been observed over the last decades [1, 2]. MetS consists of several risk factors for cardiovascular disease (CVD) and diabetes mellitus type 2 (DM2), which include abdominal obesity, dyslipidaemia, hyperglycaemia, and hypertension [3, 4]. The syndrome affects public health, through its increased risk of morbidity and mortality [5, 6]. Differences during a lifespan may affect prevalence of MetS, and a higher risk of having MetS has been observed among women along with an increasing number of children [7]. Furthermore, an increased central obesity and insulin resistance have been observed among women from pre- to post menopause [8]. However, a decreased risk of MetS among women with a history of breastfeeding has been found [7].

Fish consumption has been associated with both CVD protective effect as well as decreased CVD mortality [9–12], and particularly positive health effects of n-3 fatty acids (FA) has been investigated [13]. Fish contains a variety of nutrients such as protein, fat (especially the long, polyunsaturated marine n-3 fatty acids), vitamin D, vitamin B12, selenium and iodine [14] which may contribute to positive health implications on MetS [5, 15]. Fatty fish has a higher content of fat and different fatty acids compared to lean fish, and lean fish contains more iodine and less energy compared to fatty fish [14]. A recent review reported that few studies have investigated associations between fish consumption and MetS prevalence, but lean and fatty fish was not investigated separately in these studies [16]. Furthermore, studies have suggested that associations between fish consumption and MetS may be gender-related [16]. In the review three of the four studies that reported associations between fish consumption and MetS, found associations among men and only one study reported associations among women. Higher fish consumption has also been associated with a healthier metabolic profile, reduced WC [17], healthier lipid profile [18, 19], and reduced blood pressure level [20].

The aim of this study was to identify associations between fish consumption and MetS and its components, with particular emphasis on detecting possible differences concerning consumption of fatty and lean fish. While previous published studies have investigated fish consumption as an entity [16], this study examined fatty and lean fish separately. Our overall hypothesis is that higher fish consumption is associated with lower risk of MetS and a healthier metabolic profile.

## Methods

### Study sample

This cross sectional study uses data from the Tromsø 6 survey (2007–08) where a representative sample (*n =* 12 981) of the Tromsø population (Northern Norway) was invited to participate [21, 22]. Tromsø 6 was carried out from October 2007 to December 2008, and a total of 12 984 participants aged 30–87 years were examined. The attendance rate was 66 % [22]. This study was approved by the Regional Committee for Medical Research Ethics, South East Norway.

### Questionnaires, measurements and serum samples

Measurements were taken according to standard protocols, and participants answered questionnaires concerning demographic data (age, education, physical activity), health and diet [21, 22]. The questionnaire containing dietary data was completed at home and brought to the study site, where it was checked by a research technician for inconsistencies and incomplete data. Fish consumption was assessed using a food frequency questionnaire (FFQ), including consumption of fatty fish (e.g. salmon, trout, mackerel, herring, halibut, redfish) and lean fish (0–1 times per month, 2–3 times per month, 1–3 times per week, 4–6 times per week, 1–2 times per day).

All examinations, measurements, and laboratory work followed standardised procedures performed by trained health personnel. Systolic and diastolic blood pressure was measured using an automated device (Dinamap Pro care 300 Monitor, GE Healthcare, Norway). The cuff was chosen after the circumference of the upper arm measure, and measured on the upper right arm in a sitting position. Waist circumference was measured without outerwear, using a measuring tape, and measured across the belly button [22].

Blood samples were collected, and venipuncture was performed with participants in a sitting position. Participants were not required to fast, but only allowed to drink water and black coffee during their visits. A light tourniquet was used and released before sampling. The blood samples were sent twice daily to the Department of Laboratory Medicine, University Hospital North Norway, Tromsø, which is an accredited laboratory (ISO-standard 17025) [22].

### Metabolic syndrome

In the present study MetS was defined by the Joint Interim Societies (JIS) definition [4], where a presence of any three of five risk factors constitutes a diagnosis of MetS. For waist circumference (WC) the International Diabetes Foundation (IDF) cut points were used [23]. To diagnose MetS a presence of three or more of the five risk factors constitutes a diagnosis of MetS. The following criteria were used in this study: WC ≥94 cm in men and ≥80 cm in women. For triglycerides (TG) ≥150 mg/dL (1.7 mmol/L), for high-density lipoprotein cholesterol (HDL-C) <40 mg/dL (1.0 mmol/L) in men and <50 mg/dL (1.3 mmol/L) in women, and for glucose ≥100 mg/dL (5.5 mmol/L). For blood pressure the criteria was systolic blood pressure (SBP) ≥130 mmHg and/or diastolic blood pressure (DBP) ≥85 mmHg. The serum glucose used in this study was non-fasting.

### Statistical analyses

Due to hormonal differences in men and women, and changes over a lifespan (pre- and postmenopausal women, aging), data are presented stratified by gender and age-groups (<45 years, 45–59 years, 60–69, ≥70 years). Fish consumption was analysed both as less/higher than once a week, and as 0–1 times/month, 2–3 times/month, 1–3 times/week, 4–6 times/week and 1–2 times/day.

The MetS components were analysed as continuous variables. To investigate crude associations between fish consumption and MetS prevalence or its components, Chi-Square tests for categorical variables and analysis of variance (ANOVA) for continuous variables were used. Correlations between pairs of continuous variables were examined by Pearson’s correlation. Linear regression models were fitted to examine relationships between components of MetS (continuous) as dependent variable, and fish consumption (categorical) as independent variable. Logistic regression models were used when analysing MetS as a binary outcome variable, and potential confounding variables were adjusted for (age, physical activity, cod liver oil, parity, lactation). P-value <0.05 was considered statistical significant. All tests were two-sided. Analyses were performed using IBM SPSS Statistics 22.

## Results

The present study consisted of 12 981 participants, mean age 57.5 (range 30–87 years, 47 % men and 53 % women). Of these, 45.1 % had had acquired at least a higher education with high school diploma or more. Among women, 90.3 % reported giving birth (range 0–12 children), with an average of 2.3 (1.3) children. Among the participants, 91.4 % consumed fish (both fatty and lean) once a week or more, 72.3 % consumed lean fish and 57.1 % consumed fatty fish once a week or more. In the whole sample, those consuming fish once a week or more were significantly older, with significantly lower TG and significantly higher HDL-C, blood pressure and glucose, compared to those consuming fish less than once a week. However, when investigating the associations among men and women separately, there was not a statistically significant difference in TG level among women who consumed fish once a week or more, compared to those consuming fish less than once a week. Further, there was neither any statistically significant difference in DBP among men, for those consuming fish once a week or more, compared to those consuming fish less than once a week (Table 1).

**Table 1:** Characteristics of participants by consumption of fish, mean (SD). The Tromsø Study: Tromsø 6

		Fish consumption		
		<1/week	≥1/week	P*
Age (years)	Total	51.0 (11.87)	59.1 (12.34)	<0.0001
	Women	51.3 (12.66)	59.0 (12.59)	<0.0001
	Men	50.7 (10.93)	59.2 (12.04)	<0.0001
Waist circumference (cm)	Total	94.8 (12.32)	94.9 (12.19)	0.5
	Women	90.6 (12.16)	91.0 (12.15)	0.3
	Men	99.5 (10.68)	99.5 (10.55)	0.9
Triglycerides (mmol/l)	Total	1.63 (1.05)	1.50 (0.95)	<0.0001
	Women	1.41 (0.88)	1.39 (0.92)	0.6
	Men	1.89 (1.15)	1.63 (0.97)	<0.0001
HDL-cholesterol (mmol/L)	Total	1.44 (0.41)	1.53 (0.44)	<0.0001
	Women	1.58 (0.41)	1.66 (0.44)	<0.0001
	Men	1.28 (0.34)	1.37 (0.39)	<0.0001
Systolic blood pressure (mmhg)	Total	133.4 (22.31)	140.2 (24.2)	<0.0001
	Women	130.0 (24.30)	138.5 (26.23)	<0.0001
	Men	137.3 (19.17)	142.2 (21.50)	<0.0001
Diastolic blood pressure (mmhg)	Total	78.0 (11.61)	78.8 (11.41)	0.001
	Women	74.7 (11.44)	76.2 (11.34)	<0.0001
	Men	81.6 (10.69)	81.2 (10.74)	0.5
S-Glucose (mmol/L) ^a^	Total	5.15 (1.13)	5.26 (1.24)	<0.0001
	Women	5.02 (0.91)	5.14 (1.08)	<0.0001
	Men	5.30 (1.32)	5.40 (1.40)	0.02

Consumption of fish less than once a week/once a week or more, in total population and by gender, mean (SD)

Numbers of participants vary because of missing information variables

*WC* Waist circumference, *S-TG* S-Triglycerides, *HDL-C* High Density Lipoprotein-Cholesterol, *SBP* Systolic blood pressure, *DBP* Diastolic blood pressure

*p-value by one-way analysis of variance

^a^Serum glucose measured in Tromsø 6 was non-fasting

When modeled with linear regression models, we revealed statistically significant correlations between MetS components as dependent variables and age as independent variable. All the MetS components increased with age, except for TG which decreased among older men (B = −0.012, CI −0.015 to –0.010). Serum TG levels increased with age among women (B = 0.007, CI 0.006 to 0.009) but not men. Further, decrease in weight was correlated with increase in age among men (B = −0.19, CI −0.213 to –0.159). However, among women the decrease was smaller (B = −0.05, CI −0.074 to –0.027).

MetS prevalence was 22.6 %, with the highest prevalence of MetS among men (Table 2). The prevalence of MetS increased with age for both genders with a drop in men ≥70 years. Only 0.8 % of the participants was diagnosed with all five component of MetS, 5.7 % with four components, 16.1 % with three components, 28.8 % with two components, 34.4 % with one component and 14.2 % did not fulfil the criteria for any of the five component of MetS.

**Table 2:** Prevalence of metabolic syndrome (%) defined by the JIS definition. The Tromsø Study: Tromsø 6

	n	%
Total population	2 927	22.5
Women	1 329	19.2
<45 years	209	13.6
45 –59 years	324	17.0
60 –69 years	474	22.5
≥70 years	322	24.4
Men	1 598	26.5
<45 years	296	23.1
45 –60 years	484	28.0
60 –70 years	595	29.9
≥70 years	223	21.6

Metabolic syndrome criteria by the JIS definition: Waist circumference ≥94 cm in 21.6men and ≥80 cm in women, S-triglycerides ≥150 mg/dL (1.7 mmol/L), S-HDL cholesterol <40 mg/dL (1.0 mmol/L) in men and <50 mg/dL (1.3 mmol/L) in women, systolic blood pressure ≥130 mmHg and diastolic blood pressure ≥85 mmHg, S-glucose ≥100 mg/dL (5.5 mmol/L). Serum glucose measured in Tromsø 6 was non-fasting

### Consumption of fish and components of MetS

In linear regression models associations between consumption of fatty and lean fish (0–1 times per month, 2–3 times per month, 1–3 times per week, 4–6 times per week, 1–2 times per day) were investigated respectively as independent variables, with different components of MetS (WC, S-TG, S-HDL-C, SBP, DBP, S-Glucose) as dependent variables both in the whole sample, and among men and women individually (Table 3 and 4).

**Table 3:** Estimated change in various components of metabolic syndrome by an increasing consumption of fatty fish

		Total population			Women			Men		
		B	95 % CI	p-value	B	95 % CI	p-value	B	95 % CI	p-value
WC	Unadjusted	0.086	–0.159 to 0.330	0.5	0.130	–0.203 to 0.463	0.4	0.121	–0.191 to 0.433	0.4
	Age adjusted	–0.116	–0.360 to 0.129	0.4	–0.093	–0.435 to 0.239	0.6	–0.043	–0.354 to 0.269	0.8
S-TG	Unadjusted	–0.045	–0.063 to–0.027	<0.0001	–0.020	–0.041–0.001	0.07	–0.070	–0.099 to–0.041	<0.0001
	Age adjusted	–0.044	–0.062 to–0.025	<0.0001	–0.034	–0.056 to–0.013	0.001	–0.051	–0.080 to–0.022	0.001
HDL-C	Unadjusted	0.028	0.020 to 0.037	<0.0001	0.024	0.012 to 0.036	<0.0001	0.029	0.018 to 0.040	<0.0001
	Age adjusted	0.020	0.012 to 0.029	<0.0001	0.017	0.005 to 0.028	0.005	0.021	0.010 to 0.032	<0.0001
SBP	Unadjusted	1.085	0.615 to 1.556	<0.0001	1.486	0.788 to 2.183	<0.0001	0.682	0.072 to 1.298	0.03
	Age adjusted	–0.387	–0.806 to 0.032	0.07	–0.422	–1.009 to 0.164	0.2	–0.287	–0.862 to 0.287	0.3
DBP	Unadjusted	0.172	–0.053 to 0.397	0.1	0.296	–0.009 to 0.601	0.06	0.101	–0.210 to 0.411	0.5
	Age adjusted	–0.032	–0.257 to 0.193	0.8	–0.009	–0.310 to 0.292	0.9	0.020	–0.292 to 0.332	0.9
S-Glucose^a^	Unadjusted	0.023	–0.001 to 0.046	0.06	0.044	0.017 to 0.071	0.001	0.001	–0.039 to 0.042	0.9
	Age adjusted	0.001	–0.023 to 0.025	0.9	0.020	–0.006 to 0.047	0.1	–0.018	–0.059 to 0.022	0.4

Fatty fish (e.g. salmon, trout, mackerel, herring, halibut, redfish). Estimated change (regression coefficient B and 95 % confidence interval) in various components of metabolic syndrome (WC, TG, HDL, blood pressure, S-glucose) as the dependent variable, by an increasing consumption of fatty fish (0–1 times per month, 2–3 times per month, 1–3 times per week, 4–6 times per week, 1–2 times per day). The Tromsø Study: Tromsø 6

*WC* Waist circumference, *S-TG* S-Triglycerides, *HDL-C* High Density Lipoprotein-Cholesterol, *SBP* Systolic blood pressure, *DBP* Diastolic blood pressure

^a^Serum glucose measured in Tromsø 6 was non-fasting

**Table 4:** Estimated change in various components of metabolic syndrome by an increasing consumption of lean fish

		Total population			Women			Men		
		B	95 % CI	p-value	B	95 % CI	p-value	B	95 % CI	p-value
Lean fish										
WC	Unadjusted	0.535	0.250 to 0.819	<0.0001	0.459	0.072 to 0.846	0.02	0.430	0.068 to 0.793	0.02
	Age adjusted	–0.015	–0.309 to 0.279	0.9	–0.077	–0.473 to 0.319	0.7	–0.089	–0.468 to 0.290	0.6
S-TG	Unadjusted	–0.052	–0.075 to–0.030	<0.0001	–0.001	–0.030 to 0.028	0.9	–0.117	–0.151 to–0.083	<0.0001
	Age adjusted	–0.051	–0.074 to–0.027	<0.0001	–0.036	–0.065 to–0.006	0.02	–0.059	–0.094 to–0.023	0.001
HDL-C	Unadjusted	0.031	0.021 to 0.041	<0.0001	0.025	0.011 to 0.038	<0.0001	0.043	0.030 to 0.055	<0.0001
	Age adjusted	0.010	–0.001 to 0.020	0.06	0.008	–0.006 to 0.022	0.3	0.016	0.002 to 0.029	0.02
SBP	Unadjusted	3.810	3.266 to 4.355	<0.0001	4.520	3.713 to 5.327	<0.0001	2.916	2.210 to 3.621	<0.0001
	Age adjusted	–0.230	–0.734 to 0.275	0.4	–0.066	–0.766 to 0.635	0.9	–0.123	–0.822 to 0.576	0.7
DBP	Unadjusted	0.431	0.169 to 0.692	0.001	0.853	0.498 to 1.208	<0.0001	–0.148	–0.508 to 0.213	0.4
	Age adjusted	–0.136	–0.406 to 0.134	0.3	0.128	–0.232 to 0.487	0.5	–0.436	–0.815 to–0.058	0.02
S-Glucose^a^	Unadjusted	0.061	0.033 to 0.089	<0.0001	0.047	0.015 to 0.079	0.004	0.073	0.026 to 0.120	0.002
	Age adjusted	0.001	–0.028 to 0.030	0.9	–0.010	–0.043 to 0.022	0.5	0.011	–0.038 to 0.060	0.7

Estimated change (regression coefficient B and 95 % confidence interval) in various components of metabolic syndrome (WC, TG, HDL, blood pressure, S-glucose) as the dependent variable, by an increasing consumption of lean fish (0–1 times per month, 2–3 times per month, 1–3 times per week, 4–6 times per week, 1–2 times per day). The Tromsø Study: Tromsø 6

*WC* Waist circumference, *S-TG* S-Triglycerides, *HDL-C* High Density Lipoprotein-Cholesterol, *SBP* Systolic blood pressure, *DBP* Diastolic blood pressure

^a^Serum glucose measured in Tromsø 6 was non-fasting

An increased consumption of fatty fish was associated with a significant decrease in TG and significant increase in HDL-C, both in the whole sample and when stratified by gender. These associations remained statistically significant also after adjusting for age. An increased consumption of fatty fish was associated with a significantly higher SBP in the whole sample, however when adjusted for age this association no longer remained statistically significant. In the age adjusted model an increased consumption of fatty fish was associated with a significantly lower SBP. An increased consumption of fatty fish was also associated with significantly higher serum glucose among women; however the association did not remain significant when adjusted for age (Table 3).

An increased consumption of lean fish was associated with a significantly higher WC. However, the association was no longer statistically significant in the age adjusted model.

An increased consumption of lean fish was associated with a significant decrease in TG, both in the crude and the age adjusted model. An increased consumption of lean fish was significantly associated with an increased HDL-C, both in the whole sample and among men and women. However, this association did not remain significant in the age adjusted models for HDL-C among women. An increased consumption of lean fish was associated with a significantly lower DBP, however only among men in the age adjusted model. An increased consumption of lean fish was associated with significantly higher serum glucose; however the association did not remain significant in the age adjusted models (Table 4).

### Fish consumption and metabolic syndrome

Consumption of fish and associations with MetS as an entity was investigated using logistic regression, both in the whole sample and separately for males and females (Table 5).

**Table 5:** Fish consumption and associations with metabolic syndrome. The Tromsø Study: Tromsø 6

	Model	B	OR	95 % CI	p
Fatty and lean fish					
Total	1	–0.087	0.92	0.83 to 1.02	0.1
Women	1	0.002	1.00	0.86 to 1.17	0.9
Men	1	–0.159	0.85	0.74 to 0.98	0.03
Total	2	–0.211	0.81	0.73 to 0.90	<0.0001
Women	2	–0.184	0.83	0.71 to 0.97	0.02
Men	2	–0.206	0.81	0.70 to 0.94	0.006
Total	3	–0.191	0.83	0.74 to 0.93	0.001
Women	3	–0.203	0.82	0.68 to 0.98	0.03
Men	3	–0.162	0.85	0.73 to 0.99	0.04
Fatty fish					
Total	1	–0.019	0.98	0.90 to 1.07	0.6
Women	1	0.049	1.05	0.93 to 1.18	0.4
Men	1	–0.056	0.95	0.84 to 1.06	0.3
Total	2	–0.063	0.94	0.86 to 1.02	0.1
Women	2	–0.030	0.97	0.86 to 1.10	0.6
Men	2	–0.072	0.93	0.83 to 1.05	0.2
Total	3	–0.028	0.97	0.89 to 1.07	0.6
Women	3	–0.020	0.98	0.85 to 1.13	0.8
Men	3	–0.013	0.99	0.87 to 1.12	0.8
Lean fish					
Total	1	–0.035	0.97	0.88 to 1.06	0.5
Women	1	0.060	1.06	0.93 to 1.22	0.4
Men	1	–0.125	0.88	0.78 to 1.00	0.06
Total	2	–0.137	0.87	0.79 to 0.96	0.005
Women	2	–0.088	0.92	0.80 to 1.05	0.2
Men	2	–0.162	0.85	0.74 to 0.97	0.02
Total	3	–0.156	0.86	0.77 to 0.95	0.004
Women	3	–0.165	0.85	0.72 to 0.99	0.04
Men	3	–0.134	0.88	0.76 to 1.01	0.07

OR and p-value by Logistic regression (binary) with metabolic syndrome as dependent and frequency of fish consumption as independent (less than once a week = 0/once a week or more = 1). Model 1: Unadjusted. Model 2: Age adjusted, Model 3: Further adjusted for physical activity, cod liver oil, and parity and lactation in women. Metabolic syndrome criteria by the JIS definition: Waist circumference ≥94 cm in men and ≥80 cm in women, Triglycerides ≥150 mg/dL (1.7 mmol/L), HDL cholesterol <40 mg/dL (1.0 mmol/L) in men and <50 mg/dL (1.3 mmol/L) in women, Glucose ≥100 mg/dL (5.5 mmol/L), Systolic blood pressure ≥130 mmHg and Diastolic blood pressure ≥85 mmHg. Serum glucose measured in Tromsø 6 was non-fasting. Fish consumption (less than once a week/once a week or more). Numbers of participants vary because of missing information variables. The Tromsø Study: Tromsø 6

When investigating consumption of both fatty and lean fish together (crude model), men consuming fish once a week or more had 15 % lower risk of having MetS compared to those consuming fish less than once a week (OR 0.85, CI 95 % 0.74 to 0.98, *P =* 0.03). In the age adjusted model, a lower risk of having MetS was revealed among those consuming fish once a week or more compared to those consuming fish less than once a week. This was observed both among women (OR 0.83, CI 95 % 0.71 to 0.97, *P =* 0.02) and men (OR 0.81, CI 95 % 0.73 to 0.90, *P =* 0.006). These associations remained significant after further adjustment for physical activity, consumption of cod liver oil and parity and lactation among women.

When investigating consumption of fatty fish and lean fish separately, no significant association was found between fatty fish consumption and risk of having MetS.

When investigating associations between lean fish consumption and risk of having MetS, consumption of lean fish was significant associated with lower risk of having MetS both in the whole sample (OR 0.87, CI 95 % 0.79 to 0.96), and among men (OR 0.85, CI 95 % 0.74 to 0.97) in the age adjusted model. This association was however only borderline significant among men in the crude model.

After further adjustments (physical activity, consumption of cod liver oil and parity and lactation among women), those consuming lean fish once a week or more had a significantly lower risk of having MetS, compared to their counterparts, both in the whole sample (OR 0.86, CI 95 % 0.77 to 0.95) and among women (OR 0.85, CI 95 % 0.72 to 0.99), but not among men.

A lower risk of having MetS was found among women breastfeeding for ten months or more, compared to their counterparts (OR 0.76, CI 95 % 0.64 to 0.91) after adjusting for age and parity. In addition, parity increased the risk of having MetS, compared to their counterparts, however not significantly (OR 1.30, CI 95 % 1.03 to 1.63) in the age adjusted model.

## Discussion

### Fish consumption and metabolic syndrome

In this cross-sectional study higher fish consumption was associated with a lower risk of having MetS. When investigating fatty and lean fish separately, only lean fish consumption was associated with lower risk of having MetS. A few other studies have also reported associations between consumption of fish and MetS [18, 19, 24, 25]. In a large Korean follow-up study (*n =* 3504) they found that the risk of having MetS decreased among men who consumed fish daily, compared to those consuming fish less than once a week (OR 0.43, 95 % CI 0.23 –0.83) [18]. In this study associations between fish consumption (sum of dark- or white-meat fish and canned tuna) and incidence of MetS was investigated, among participants aged 40–69 years without MetS at baseline. They did, however, not find any association among women. This study defined MetS according to the Adult Treatment Panel III (ATP III) definition [23], except for WC where alternative criteria were used [26]. Associations between fish consumption and MetS have also been observed in previous cross-sectional studies [19, 24, 25]. To the best of our knowledge, only one study found associations between consumption of fish and MetS among women, indicating that there might be gender differences [19]. However, not all studies find associations between consumption of fish and lower MetS prevalence [27, 28].

In this study, lean fish consumption in particular seems to be associated with a lower risk of MetS and its components. Lean fish, such as cod, are considered a superior source of proteins, and have been associated with reduction in body weight [29]. This effect may be due to their positive effect on satiety, which has been observed in a study comparing fish protein with other animal proteins [30]. Dietary proteins regulate lipid metabolism, and have been seen to slow absorption and synthesis of lipids, and promote the lipid excretion [31]. Fish proteins are easily digestible and rich in essential amino acids, and animal studies suggest that fish protein may have effects on both plasma and liver lipids [32]. Furthermore, consumption of proteins from fish might have beneficial effects on hyperglycaemia and hyperlipidaemia [33, 34]. An improved insulin sensitivity in insulin-resistant men and women consuming proteins from cod have also been observed, when compared to other animal proteins [35].

### Gender differences in prevalence of metabolic syndrome

In this study the overall MetS prevalence was 23 %, and prevalence of MetS increased with age in line with other studies [36]. One exception was men in the highest age group (≥70 years), where the prevalence of MetS decreased. A decreased TG along with an increased age was found among men in this study. In men, aging is associated with a decline in testosterone [37], which has been associated with increased prevalence of obesity [38] and MetS [37]. Low testosterone levels have also been associated with higher TG levels among men [39]. However, men with normal weight seems to have higher testosterone levels, compared to obese men [37]. In this study a decrease in weight along with an increasing age among men was observed, which may have affected the lower prevalence of MetS in the oldest age groups in this study.

Previous evidence has indicated a 20 –30 % prevalence of MetS among the adult population in most countries [40]. Other studies have also identified lower prevalence in a young population [19], and higher prevalence among elder populations [28]. In this study, women ≥70 years had a slightly higher prevalence of MetS, compared to men in the same age group. This is in line with other studies, that also found higher prevalence of MetS among women, compared to men [41, 42]. Differences between men and women that occur during a lifespan may affect prevalence of MetS. Previously, both parity and an increasing number of children have been associated with higher risk of having MetS [7], and especially an increased central obesity have been observed in the transition from pre- to post menopause in women [8]. In this study, women also had a somewhat higher WC than men according to the MetS criteria for WC. Also insulin resistance and a more atherogenic lipid profile (decreased HDL-C, increased TG and low density lipoprotein) among women have been associated with the transition from pre- to post menopause [8]. However, increased TG levels with increasing age have been reported both in men and women [43]. Both testosterone levels and oestrogen levels changes over a lifespan, and have been associated with components of MetS [43]. Women also have higher HDL-C than men in all ages [43], which is reflected in the MetS definition [4]. Previously, a decreased risk of MetS among women with a history of breastfeeding has been suggested [7, 44]. In this study, fish consumption was associated with a lower risk of having MetS only among men in the crude model. However, after adjusting for parity and lactation among women in the multi adjusted models, associations between fish consumption and a lower risk of having MetS among women was observed. Further, lean fish seemed to be responsible for this association. In this study, women with a longer duration of lactation had lower risk of MetS. Lactation imposes a metabolic burden on women, due to the increased energy requirement [45, 46], and changes that occur during pregnancy such as increased visceral fat, insulin resistance and increased TG levels, may reverse more quickly and more completely with lactation [44, 47, 48]. Both parity and lactation may influence the results, and should therefore be adjusted for.

### Fish consumption and components of metabolic syndrome

In this study a higher consumption of fish was associated with a decreased TG, this in line with previous studies. Both in a prospective cohort study (*n =* 3 504) [18], and in a cross-sectional studies consisting of women [19, 49]. However, higher consumption of fish has also been associated with higher TG level [27]. Nonetheless, none of these studies investigated fatty and lean fish separately. In this study, higher consumption of fatty as well as lean fish was associated with a decreased TG. A randomized controlled trial from Norway, investigated if consumption of fatty (salmon) and lean (cod) could influence TG level among healthy adults (*n =* 30), and found that both fatty and lean fish decreased TG level significantly [50]. Also another randomized controlled trial recently found reduced serum TG among those consuming lean seafood, when investigating dietary protein sources ingested from lean seafood (cod) and non-seafood in a four weeks lean-seafood intervention [51].

In this study a higher consumption of fish was also associated with an increased HDL-C, in line with previous studies [18, 19]. However, the prospective Korean study found this association only among men [18], and the Iranian cross sectional study found this association in a population consisting of only women [19]. Further, none of these studies investigated fatty and lean fish separately, which was done in this study. However, no association was found between consumption of lean fish and HDL-C level in the Norwegian intervention study [50], who found a significant increase in HDL-C among those consuming fatty fish [50]. A low HDL-C level may be a pre-existing phase of MetS [52], and there has been observed that adults with low HDL-C level were more susceptible to developing MetS over time in a five year follow-up (*n =* 4 905) [52].

In agreement with other studies [18, 19], the present study did not find any association between fish consumption and WC after adjusting for age. In contrast, one intervention study investigating the effect of lean fish on CV risk factors in patients with MetS found that consumption of fish was associated with a reduced WC [17], indicating that this association is inconclusive.

In the present study an increasing consumption of fatty fish was associated with a significant lower SBP both in total population and among men and women, and consumption of lean fish was associated with a reduction in DBP among men. Higher fish consumption has previously also been associated with decreased blood pressure [19, 20]. However, some studies only found associations between consumption of fish and a lower DBP [17].

In the present study higher consumption of fish was associated with increased glucose level, but the association did not remain significant after adjusting for age. However, the serum samples used in this study are non-fasting, which may have affected the results.

### Strengths and limitations

The main strength of this population-based study is a large sample size. Moreover, all examinations, measurements, and laboratory work followed standardised procedures performed by trained health personnel [25]. However, associations in this study are investigated cross-sectional and can therefore not provide insights on causation between fish consumption and MetS. This study has no information on cooking method, and any positive health effects may diminish or vanish depending on how the meal is prepared. Furthermore, there is an overlap between fatty fish consumption and lean fish consumption, which may affect the result. Serum samples are non-fasting, and may therefore be less accurate according to the MetS definition.

## Conclusions

In this study based on an adult population from Northern Norway, higher fish consumption and particularly lean fish consumption, was associated with a lower risk of having MetS. Moreover, both higher fatty fish consumption and lean fish consumption were associated with a decreased TG and an increased HDL-C. Further investigation is warranted to establish associations between fish consumption and MetS and its components, in particular with regard to further explore possible differences in how fatty and lean fish consumption may influence MetS risk.

### Availability of data and materials

Available variables from the Tromsø Study (Tromsøs 1–6) may be viewed at the website http://www.tromsostudy.com.
